# Preoperative magnetic resonance imaging predicts clinicopathological parameters and stages of endometrial carcinomas

**DOI:** 10.1002/cam4.4486

**Published:** 2021-12-30

**Authors:** Chia‐Ying Wu, Yi‐Jou Tai, I‐Lun Shih, Ying‐Cheng Chiang, Yu‐Li Chen, Heng‐Cheng Hsu, Wen‐Fang Cheng

**Affiliations:** ^1^ Department of Obstetrics and Gynecology College of Medicine National Taiwan University Taipei Taiwan; ^2^ Department of Obstetrics and Gynecology National Taiwan University Hospital Taipei Taiwan; ^3^ Department of Medical Imaging College of Medicine National Taiwan University Taipei Taiwan; ^4^ Graduate Institute of Clinical Medicine College of Medicine National Taiwan University Taipei Taiwan; ^5^ Department of Obstetrics and Gynecology National Taiwan University Hospital Xin‐Chu City Taiwan; ^6^ Graduate Institute of Oncology College of Medicine National Taiwan University Taipei Taiwan

**Keywords:** endometrial carcinoma, magnetic resonance image, metastasis, stage

## Abstract

**Background:**

We investigated the agreement and accuracy of preoperative magnetic resonance imaging (MRI) with postoperative pathological characteristics and stages of endometrial endometrioid carcinoma (EEC).

**Methods:**

We recruited 527 women with EEC who underwent staging surgery at a single medical institution. The preoperative MRI, stages, and clinical and pathological parameters, including myometrial invasion (MI), cervical invasion (CI), adnexal metastasis (AM), intra‐abdominal metastasis, and pelvic and/or para‐aortic nodal metastasis, were recorded and analyzed. The agreement and accuracy between the preoperative MRI findings and these parameters and stages were assessed.

**Results:**

The rate of the preoperative MRI‐based clinical stage matching the postoperative surgical stage was 85.2% in International Federation of Gynecology and Obstetrics stage IA, 51.9% in stage IB, 35.5% in stage II, 5.3% in stage IIIA, 33.3% in stage IIIB, 28.6% in stage IIIC1, 64.3% in stage IIIC2, and 93.8% in stage IVB. The consistency between radiologists and pathologists was 80.5% for deep MI, 91.5% for cervical invasion, 92.2% for adnexal metastasis, 98.9% for intra‐abdominal metastasis, and 87.5% and 92.2% for pelvic and para‐aortic nodal metastases, respectively. The negative predictive value of intra‐abdominal metastasis was the highest with 99.8%.

**Conclusions:**

Preoperative MRI could be an excellent tool for routine preoperative assessment to predict pathological parameters and stages of EEC, especially in excluding intra‐abdominal metastatic disease.

## Graphical abstract

Preoperative magnetic resonance imaging (MRI) had good correlations with the pathologic parameters and stages of endometrial endometrioid adenocarcinoma. Preoperative MRI had high NPVs for disease extent, particularly the extra‐uterine spread. Preoperative MRI could provide an excellent tool for routine preoperative assessment of endometrial carcinoma patients.

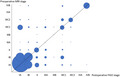

## 1 INTRODUCTION

Uterine cancer is the sixth most common cancer in women worldwide with about 417,000 women newly diagnosed in 2020.^1^ It is also the fourth most common cancer in the United States, with data from the U.S. National Cancer Institute’s Surveillance, Epidemiology, and End Result program indicating that new uterine cancer cases have increased an average 0.5% annually during the last 10 years.^2^, ^3^, ^4^ The incidence of uterine cancer in Taiwan was 24.7 per 100,000 women according to the nationwide population‐based Taiwan Cancer Registry in 2018.^5^ In Taiwan, uterine cancer was not in the top 10 cancers prior to 2006,^6^, ^7^ but its incidents have been rapidly increasing since 2000.^8^

Uterine cancers can be categorized as carcinomas, carcinosarcomas, or sarcomas. Approximately 86% of uterine cancers are carcinomas.^5^, ^9^ About 80%–90% of endometrial cancers, also called endometrial carcinomas (ECs), are endometrioid type.^10^ The majority of ECs is diagnosed in the initial stages because the main symptom of abnormal or menopausal vaginal bleeding appears early in the disease and prompts investigation. The endometrioid carcinomas were regarded as type I uterine carcinomas. Uterine papillary serous and clear cell carcinoma were regarded as type II uterine carcinomas which had different treatment strategies and outcomes compared with those type I uterine carcinomas. The International Federation of Gynecology and Obstetrics (FIGO) Committee shifted from clinical to surgical staging of EC in 1988 and revised the staging system in 2009.^11^ A standard staging procedure, including hysterectomy, bilateral salpingo‐oophorectomy, extrauterine tumor excision, and selective pelvic and/or para‐aortic lymph node dissectionis routinely recommended for uterine cancer patients.^12^ Hysterectomy with/without bilateral salpingo‐oophorectomy alone would be an alternative for low‐risk patients based on the imaging results or those who could not tolerate longer operative time.

Because staging depends on postoperative findings, the estimated stage and risk of extrauterine disease determines the extent of surgery. Extrauterine spread, the depth of myometrial invasion (MI), tumor grade and histological subtype, and lymph node metastases are the factors considered in endometrial cancer prognosis.^12^ Complete lymph node dissection is associated with morbidity including lymphedema, lymphocele formation, and neuralgia.^13^ An international consensus conference generated the recommendation that the indication for lymph node dissection should stratify the cases to low, intermediate, or high risk.^12^ Currently, preoperative magnetic resonance imaging (MRI) and/or computerized tomography (CT), intraoperative frozen section and sentinel lymph node mapping could be used if full staging is needed.^14^ The GOG 99 and PORTEC trials defined risk factors for those at high to intermediate risk of recurrence, including MI on preoperative imaging and intraoperative surgical findings. Low risk for nodal metastasisis characterized by <50% MI, tumor size <2 cm, and well to moderately differentiated histology.^15^ One retrospective analysis of low‐risk patients without lymphadenectomy reported no significant difference in overall survival and progression‐free survival compared to those who underwent lymphadenectomy.^16^ Evidence on the accuracy of the currently available tools (especially imaging) remain lacking in literature.^17^, ^18^, ^19^, ^20^, ^21^, ^22^, ^23^, ^24^, ^25^, ^26^, ^27^ Therefore, the optimal selection of patients who can avoid lymph node dissection and the determination of the extent of lymphadenectomy for high‐ to intermediate‐risk patients remain clinical challenges.^28^, ^29^

The guidelines recommend MRI for estimating the preoperative stage because of the better resolution of soft tissue contrast for assessing the depth of myometrial or cervical invasion compared to CT.^12^, ^30^, ^31^, ^32^ Ultrasound relies on operator expertise. Positron emission tomography (PET) is not yet widely used for preoperative evaluation in EC due to cost and inaccessibility.

Several studies have reported the assessment of preoperative image reliability, but numbers of patients are limited and not all risk factors were discussed.^24^, ^25^, ^26^, ^28^ So, we conducted a retrospective study to evaluate the diagnostic performance of preoperative MRI in EC staging in routine clinical practice.

## 2 MATERIALS AND METHODS

### 2.1 Patients

A total of 1020 patients with EC were identified from the National Taiwan University Hospital covering the period from January 1, 2013, to December 31, 2018. This study was approved by the Institutional Research Ethics Committee at the National Taiwan University Hospital. All of the patients’ data were fully anonymized before we accessed them and the Research Ethics Committee waived the requirement for informed consent. All patients were diagnosed by endometrial biopsy or curettage, with confirmation by hysterectomy. We excluded cases in which patients did not undergo hysterectomy because of personal reasons, were not available for preoperative MRI at our hospital, were not good candidates for surgery, had undergone surgery at another hospital, had incidental cancer such as ovarian cancer after the hysterectomy, or had undergone surveillance at the other hospitals after surgery. We also excluded 66 patients with other histological types, including serous and clear cell carcinoma, adenosarcoma, carcinosarcoma, leiomyosarcoma, and neuroendocrine carcinoma. Data of the remaining 527 patients were eligible for further analysis (Figure S1).

### 2.2 MRI examinations

All of the 527 patients underwent abdomino‐pelvic MRI to examine upper abdomen. The chest CT scan was only performed when suspected pulmonary metastasis by CxR or clinical symptoms such as cough. MRI examinations were performed using a 1.5‐T MRI unit (SignaHDx; GE Healthcare). The pulse sequences for pelvic imaging included T2‐weighted fast spin echo (FSE) sequences in the sagittal, coronal oblique, and axial oblique views according to the axis of the uterine body, an axial T2‐weighted FSE sequence with fat saturation (FS) of the whole pelvis, an axial T1‐weighted gradient‐echo (GRE) sequence with FS, and an axial diffusion‐weighted echo‐planar imaging (DW‐EPI) sequence (*b*‐values, 0, and 800 s/mm^2^) for the whole pelvis. Examinations performed after 2018 also included a sagittal DW‐EPI of the uterus. Apparent diffusion coefficient (ADC) maps were derived from the diffusion‐weighted sequences, generated by the scanner software. The patients received intravenous gadolinium contrast medium (0.1 mmol/kg of gadoterate meglumine, Dotarem; Guerbet) if there were no contraindications. Post‐contrast images include T1‐weighted three‐dimensional‐spoiled GRE sequence with FS in the sagittal, coronal, and axial views. Images of the upper abdomen were also obtained to detect possible metastases. The pulse sequences for upper abdomen include an axial T1‐weighted GRE sequence with FS, an axial T2‐weighted FSE sequence with FS, and post‐contrast T1‐weighted three‐dimensional‐spoiled GRE sequence with FS in the coronal and axial views. The details of these pulse sequences are summarized in Table 1. The MRI examinations were interpreted by total 11 well‐experienced and qualified radiologists, who are familiar with abdominal and pelvic imaging. The imaging reports were obtained from the electronic medical record of the hospital. We recorded the following findings from the imaging reports including MI, CI, AM, pelvic and/or para‐aortic lymph node metastases, and intra‐abdominal metastases. MI was defined as abnormal signal intensity of the tumor extended into the myometrium. CI was defined as disruption of the hypointense cervical stroma by the tumor. AM was defined as abnormal mass involving the adnexa. Lymph nodes with a short axis >1 cm, or with suspicious features including multiple small rounded nodes, irregular contour, abnormal signal intensity similar to that of primary tumor, or presence of necrosis, were considered to be nodal metastasis.^32^, ^33^ The definition of intra‐abdominal metastasis was tumor lesions which were not included in the other five parameters including para‐aortic lymphadenopathy above the renal vessels, peritoneal metastasis such as enhancing omental or peritoneal nodules, or hepatic metastasis as hepatic nodules with mild hyperintensity on T2‐weighted images with hypoenhancement on post‐contrast images.^32^

**TABLE 1:** The relevant methods and conditions of MRI examination

	Plane	Repetition time (TR) (ms)	Echo time (TE) (ms)	Flip angle (°)	Slice thickness (mm)	Matrix	Field of view (mm)
Pelvis							
T2‐weighted FSE	Sagittal Coronal oblique Axial oblique	3500–5500	80–100	—	3–4	256 × 192	240–250
T2‐weighted FSE with FS	Axial	3500–5500	80–100	—	5–6	288 × 192	260–280
T1‐weighted GRE with FS	Axial	150	4.2	70	5–6	256 × 192	260–280
DW‐EPI	Axial	7000–9000	60–80	—	5–6	64 × 128	280–300
DW‐EPI	Sagittal	7000–9000	60–80	—	3–5	64 × 128	240–250
Post‐contrast T1‐weighted GRE with FS	Axial Sagittal Coronal	3.8–4.6	1.8–2.3	15	3–4	288 × 160	240–260
Abdomen							
T1‐weighted GRE sequence with FS	Axial	150	4.2	80	5–6	256 × 192	300–320
T2‐weighted FSE sequence with FS	Axial	2600–3000	80–100	—	5–6	256 × 192	300–320
Post‐contrast T1‐weighted GRE with FS	Axial Coronal	3.8–4.6	1.8–2.3	15	3–4	288×160	280–320

All 527 patients underwent complete surgical staging, including washing cytology, total hysterectomy, bilateral salpingo‐oophorectomy, pelvic and/or para‐aortic lymph node sampling or dissection, and omental biopsy. Omentectomy was only performed when intra‐abdominal metastases were suspected before or during surgery. Resection of any suspicious lesions, such as peritoneal biopsy or bowel resection was performed if indicated. Thirteen patients elected to preserve the ovaries because of their age younger than 45 years and without suspicious of malignancy before and during surgery. Staging and histological grade were postoperatively determined based on the 2009 FIGO staging system.^12^

### 2.3 Statistical analysis

Using standard statistical formulas, we calculated the accuracy, sensitivity, specificity, PPV, NPV, positive likelihood ratio (LR+), negative likelihood ratio(LR−), and kappa of MRI for determining the clinicopathological parameters. LR+ is the probability that a parameter of interest that is present was detected on MRI (true positive) divided by the probability that a parameter that is not present was detected on MRI (false positive). The higher the LR+, the more useful the positive finding will be considered. Conversely, LR− is equivalent to the probability that a person with the parameter had a negative result for it on MRI (false negative) divided by the probability that a person without this parameter tested negative for it (true negative).^34^, ^35^ The kappa statistic is a measure of agreement between radiologist‐reported MRI findings and the pathologists’ conclusions. A kappa value of zero indicates that the two results were not in agreement any more than chance alone would predict.^36^ Kappa result interpreted as Landis and Koch scale that 0.01–0.20 is none to slight agreement, 0.21–0.40 is fair, 0.41–0.60 is moderate, 0.61–0.80 is substantial, and 0.81–1.00 is almost perfect agreement.^36^

## 3 RESULTS

### 3.1 Patient characteristics

The characteristics of the 527 patients are provided in Table 1. The median age was 56.1 years (range: 28–89 years). The premenopausal patients were 189 (35.9%), and the remaining patients (*n* = 338, 64.1%) were post‐menopausal. Overall, 409 (77.6%) patients presented with FIGO stage I, 31 (5.9%) with stage II, 71 (13.5%) with stage III, and 16 (3.0%) with stage IVB disease. A total of 517 (98.1%) and 10 (1.9%) patients had endometrioid histology and endometrioid with other histologic types, respectively. Histological grade 1 was most common (*n* = 357, 67.7%), followed by grade 2 (*n* = 91, 17.3%) and grade 3 (*n* = 79, 15.0%). Of the whole cohort, 55 patients (10.4%) had malignant cells and 38 (7.2%) had cells with atypia of undetermined significance in their washing cytology or ascites. Pelvic lymph node sampling or dissection was performed in 98.3% of patients and para‐aortic lymph node sampling or dissection was in 24.3% of patients (Table 2).

**TABLE 2:** Clinico‐pathologic characteristics of 527 EEC women

Clinico‐pathologic characteristics	Patient number	%
FIGO stage^a^		
I	409	77.6
IA	332	63.0
IB	77	14.6
II	31	5.9
III	71	13.5
IIIA	19	3.6
IIIB	3	0.6
IIIC1	35	6.6
IIIC2	14	2.7
IVA	0	0
IVB	16	3.0
Histologic type		
Endometrioid	517	98.1
Mixed endometrioid and the other type^b^	10	1.9
Grade		
I	357	67.7
II	91	17.3
III	79	15.0
Cytology		
Negative	406	77.0
Positive	55	10.4
Atypia of undetermined significance	38	7.2
N/A	28	5.3
Pelvic lymph node sampling/dissection		
Yes	518	98.3
No	9	1.7
Para‐aortic node sampling/dissection		
Yes	128	24.3
No	399	75.7

Abbreviations: EEC, Endometrial endometrioid carcinoma; N/A, not available.

^a^ According to FIGO stage 2009.

^b^ Including mixed with clear cell, mucinous, serous, neuroendocrine carcinoma, and dedifferentiated carcinoma.

### 3.2 Clinical parameters detected by preoperative MRI

**FIGURE 1:**
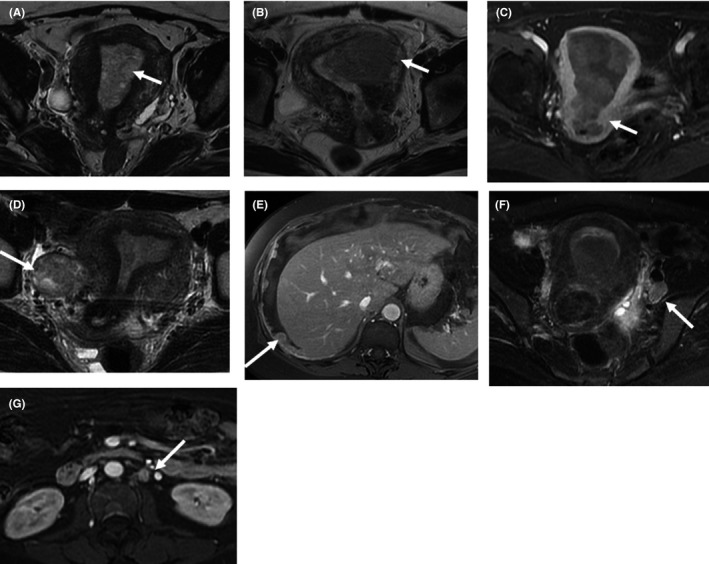
Magnetic resonance imaging of different pathological parameters. (A) Coronal oblique T2‐weighted image of the uterus showing endometrial cancer with superficial myometrial invasion in a 47‐year‐old woman. The endometrial tumor exhibits <50% myometrial invasion (arrow). (B) Coronal oblique T2‐weighted image of the uterus showing endometrial cancer with deep myometrial invasion in a 60‐year‐old‐woman. The endometrial tumor exhibits ≥50% myometrial invasion (arrow). (C) Axial post‐contrast T1‐weighted image of endometrial cancer with cervical invasion (arrow) in a 69‐year‐old woman. (D) Coronal oblique T2‐weighted image of the uterus showing endometrial cancer with adnexal metastasis in a 33‐year‐old woman. The endometrial tumor (arrow) is shown with a solid right adnexal tumor (arrowhead). (E) Axial post‐contrast T1‐weighted image of endometrial cancer with intra‐abdominal metastases in a 50‐year‐old woman with moderate ascites. Note: The multiple peritoneal tumors at the perihepatic region (arrows). (F) Axial T2‐weighted image with fat saturation of endometrial cancer with pelvic lymph node metastases in a 37‐year‐old woman. Note: The enlarged lymph node at the left external iliac region (arrow). (G) Axial post‐contrast T1‐weighted image of endometrial cancer with para‐aortic lymph node metastases in a 63‐year‐old woman. Note: The enlarged lymph node at the para‐aortic region (arrow)

Regarding the preoperative MRI findings, we analyzed six parameters of interest, including MI, CI, AM, intra‐abdominal metastasis, and pelvic and/or para‐aortic nodal metastasis (Table 3). Of the 527 patients, 29.0% (*n* = 153) had ≥50% MI, 11.8% (*n* = 62) had CI, 7.4% (*n* = 39) had AM, and 2.3% (*n* = 12) had intra‐abdominal metastasis. Pelvic lymph node metastases were identified in 54 patients (10.2%) and para‐aortic lymph node metastases in 16 patients (2.9%). Figure 1 shows preoperative MRI of MI (Figure 1A,B), CI (Figure 1C), AM (Figure 1D), intra‐abdominal metastasis (Figure 1E), and pelvic (Figure 1F) and para‐aortic (Figure 1G) nodal metastases.

**TABLE 3:** Preoperative MRI findings of 527 EEC women

	Pathologic report					
	Myometrial invasion ≥50%	Cervical stromal invasion	Adnexal metastasis	Intra‐abdominal metastasis	Pelvic nodal metastases	Para‐aortic nodal metastases
MRI assessment						
IA	29	10	4	1	4	2
IB	55	6	6	0	9	0
II	12	13	5	0	3	0
IIIA	6	3	1	0	2	1
IIIB	2	3	1	0	1	0
IIIC1	18	10	5	0	11	2
IIIC2	15	9	6	0	11	7
IVB	16	8	11	11	13	4
Total	153	62	39	12	54	16

Abbreviation: EEC, endometrial endometrioid carcinoma.

### 3.3 Correlations between MRI‐based clinical stages and surgical stages

We also evaluated the agreement between MRI‐based clinical stages and surgical stages (Table 4). The top two highest rates of agreement were FIGO stage IVB (93.8%; 15/16) followed by IA (85.2%; 283/332). The lowest rate of agreement was found on FIGO stage IIIA (5.3%; 1/19). Understaging by preoperative MRI was most common for patients with FIGO stage IIIA disease (adnexal metastases) (68.4%; 13/19), and overstaging by preoperative MRI was most common for patients with FIGO stage IIIB disease (vaginal metastasis) (33.3%; 1/3).

**TABLE 4:** Correlations between clinical stages by preoperative magnetic resonance image and postoperative surgical stages

	FIGO stage									
	IA	IB	II	IIIA	IIIB	IIIC1	IIIC2	IVA	IVB	Total
MRI stage										
IA	**283**	26	10	4	0	3	2	0	1	329
IB	31	**40**	5	5	1	9	0	0	0	91
II	2	2	**11**	4	0	3	0	0	0	22
IIIA	1	2	2	**1**	0	1	1	0	0	8
IIIB	0	0	1	0	**1**	1	0	0	0	3
IIIC1	12	3	2	4	1	**10**	2	0	0	34
IIIC2	2	2	0	1	0	6	**9**	0	0	20
IVA	0	0	0	0	0	0	0	**0**	0	0
IVB	1	2	0	0	0	2	0	0	**15**	20
Total	332	77	31	19	3	35	14	0	16	527

Bold means consistent numbers between MRI stage and FIGO stage.

### 3.4 Performance of preoperative MRI indetecting pathological parameters

Table 5 shows the performance of preoperative MRI in detecting the pathological parameters. The sensitivity, specificity, PPV, and NPV for MI ≥50% on preoperative MRI were 60.8%, 88.5%, 68.4%, and 84.7%, respectively. The results for LR+ showed that, when the preoperative MRI revealed MI ≥50%, a patient would be 5.3‐times as likely to have deep MI (≥50%) than if this parameter was not detected on MRI. In contrast, a negative result on MRI was likely 40% (LR−, 0.4) of the time in a patient with deep MI compared to a patient without it. The overall accuracy for deep MI was 80.5%, with good consistency between radiologists and pathologists. The degree of underestimating and overestimating deep MI on preoperative MRI was 11.4% and 8.2%, respectively. The overestimating rate was 15.3% (79/511) from stage IA to IIIC2.

**TABLE 5:** The measurements of the reliability of MRI in 527 EEC women

	MI ≥50%	CI	AM	IAM	PLNM	PaLNM
Sensitivity (%)	60.8	53.2	25.6	91.7	46.3	68.8
Specificity (%)	88.5	96.6	97.5	99.0	92.2	95.5
PPV (%)	68.4	67.4	45.5	68.8	41.0	68.8
NPV (%)	84.7	93.9	94.3	99.8	93.7	95.5
LR+	5.3	15.5	10.4	93.7	6.0	15.4
LR−	0.4	0.5	0.8	0.1	0.6	0.3
Kappa	0.51	0.55	0.29	0.78	0.36	0.64
Accuracy rate (%)	80.5	91.5	92.2	98.9	87.5	92.2
Overestimation (%)	8.2	3.0	2.3	1.0	7.0	3.9
Underestimation (%)	11.4	5.5	5.5	0.2	5.6	3.9

Abbreviations: AM, adnexal metastasis; CI, cervical stromal invasion; EEC, endometrial endometrioid carcinoma; IAM, intra‐abdominal metastasis; LR−, negative likelihood ratio; LR+, positive likelihood ratio (LR+); MI, myometrial invasion; N/A, not available; NPV, negative predict value; PaLNM, para‐aortic nodal metastases; PLNM, pelvic lymph nodal metastases; PPV, positive predict value.

### 3.5 Sensitivity and specificity of preoperative MRI in predicting various pathological parameters

The sensitivity, specificity, PPV, and NPV of predicting CI were 53.2%, 96.6%, 67.4%, and 93.9%, respectively. The LR+ was 15.6 (53.2%/3.4%), suggesting that when preoperative MRI showed CI, the patients would be 15.6‐times more likely to have CI than the patients in whom imaging did not show CI. The LR− was 0.48 (46.8%/96.6%), indicating that a negative result had an approximately 50% chance of being adjudicated in a person with CI as in a person without it. The agreement for CI between radiologists and pathologists was 91.5%, and the degree of underestimating and overestimating CI on preoperative MRI was 5.5% and 3.0%, respectively.

The sensitivity, specificity, PPV, and NPV of predicting AM were 25.6%, 97.5%, 45.5%, and 94.3%, respectively. The LR+ was 10.2 (25.6%/2.5%), indicating that when the preoperative MRI revealed AM, a patient would be 10.2‐times more likely to have AM than when imaging did not show it. The LR‐ was 0.76 (74.4%/97.5%); therefore, a negative result carried an 80% chance that someone with AM on MRI would have AM compared to a person without it. Radiologists and pathologists had 92.2% agreement on this parameter. The degree of underestimating and overestimating AM on preoperative MRI were 5.5% and 2.3%, respectively.

The sensitivity, specificity, PPV, and NPV of predicting intra‐abdominal metastases were 91.7%, 99.0%, 68.8%, and 99.8%, respectively. The LR+ was 93.6 (91.7%/0.98%), indicating that when the preoperative MRI revealed intra‐abdominal metastasis, a patient would be 93.6‐times more likely to have intra‐abdominal metastasis than when imaging did not show it. The LR− was 0.084 (8.33%/99.02%). Radiologists and pathologists had an accuracy of 98.9% when identifying intra‐abdominal metastases. The degree of underestimating and overestimating intra‐abdominal metastases on preoperative MRI were 0.2% and 1%, respectively. There were four clinical stage IVB patients with the metastatic lesions in supraclavicular node, bone, and labia which were excluded in the statistical analysis due to the outfield of abdomino‐pelvic MRI examination. Omental metastasis was the most common intra‐abdominal lesion in all stage IVB patients (50%, 8/16 cases) following by the intestine metastases (31.3 %, 5/16 cases). There were two patients with omental and intestinal metestases.

The sensitivity, specificity, PPV, and NPV of predicting pelvic nodal metastasis in patients who underwent pelvic lymph node dissection were 46.3%, 92.2%, 41.0%, and 93.7%, respectively. The LR+ was 5.9 (46.3%/7.8%), and the LR− was 0.58 (53.7%/92.2%). The agreement between radiologists and pathologists in identifying pelvic nodal metastasis was 87.5%. The degree of underestimating and overestimating pelvic nodal metastasis on preoperative MRI were 5.6% and 7.0%, respectively.

The sensitivity, specificity, PPV, and NPV for para‐aortic nodal metastasis in 128 patients who underwent para‐aortic lymph node dissection were 68.8%, 95.5%, 68.8%, and 95.5%, respectively. When the preoperative MRI revealed para‐aortic nodal metastasis, the patients were15.3‐times likely to have para‐aortic nodal metastasis compared with those without (LR+, 15.3; 68.8%/4.5%). The LR− was 0.33 (31.2%/95.5%). The accuracy of determining para‐aortic nodal metastasis was 92.2%, with good agreement between radiologists and pathologists, and the degree of underestimating and overestimating this parameter on preoperative MRI was 3.9% and 3.9%, respectively.

### 3.6 Influences of different radiologists and years on the interpretation of MRI results

We further evaluated if the accuracies of various pathologic factors between preoperative MRI and pathologic reports were influenced by different years or radiologists. The accuracies of various pathologic parameters detected by the radiologist who interpreted the most patient number and the other patients interpreted by the rest 10 radiologists are shown in Table S1A,B. Only the accuracy of MI ≥1/2 depth was different (85.4% vs. 74.3%, *p* = 0.0014, *Z*‐test). The accuracies of the other parameters were no different. Whereas, the accuracies of various parameters between 2013 and 2018 were no different (Table S2A,B).

Our results revealed that the interpretation of different parameters by preoperative MRI could be relied by well‐trained and experienced radiologists.

## 4 DISCUSSION

MRI is a good tool in detecting the extent of tumor in the body of uterus and cervix, adnexa, intra‐abdominal metastasis, and pelvic and/or para‐aortic nodal metastasis before definitive treatment of endometrial cancer.^21^ Our results indicated that more than 93% NPV of MRI for the five parameters except MI. Both of the PPV and NPV of MRI for intra‐abdominal metastasis were exceptionally high compared to those for the other five parameters.

A Nationwide Surveillance in Taiwan showed that the mean age of endometrial endometrioid adenocarcinoma women was 53 years old which was consistent with our observation but was younger than that of women with clear cell carcinoma, uterine serous carcinoma, or carcinosarcoma.^37^ The percentages of newly‐diagnosed uterine cancer in Taiwan were 70.9% with stage I, 5.4% in stage II, 13.4% in stage III, and 7.8% in stage IV, according to the nationwide population‐based Taiwan Cancer Registry in 2018.^5^ The stage distribution in our study was similar to the result of the nationwide surveillance.

Preoperative MRI is one of the evaluation tools for staging EC.^30^ One risk factor associated with lymph node invasion is MI. On T2‐weighted images, endometrial cancer tumor appears as intermediate signal intensity, and disruption of the junctional zone was interpreted as MI. The pitfalls in the assessment of MI have been associated with the presence of leiomyoma, adenomyosis, poor tumor to myometrium contrast, loss of the junctional zone definition, and extension of the tumor into the cornua.^20^, ^32^ Wu et al.^38^ reported that preoperative MRI imaging has a sensitivity, specificity, PPV, and NPV of 92.5%, 74.3%, 71.4%, and 93.5%, respectively, for the identification of deep MI. In our study, the patterns were consistent with previous results,^39^ with a lower false‐negative rate than true‐positive rate. Body et al. reported a sensitivity of 73.7% and specificity of 88.0% for the deep of MI.^39^ Because the revised staging system by FIGO in 2009 omitted cervical mucosal invasion and kept the cervical stromal invasion as stage II disease, so radiologists could interpret CI according to morphologic imaging by DWI and dynamic contrast‐enhanced image. Using these imaging techniques, the accuracy of diagnosing stage II disease increased since then.^21^ Using T2‐weighted MRI and DCE‐MRI, Lin et al.^40^ found an accuracy, sensitivity, and specificity of 87%, 58%, and 95%, respectively. They also showed that DWI could markedly improve diagnostic accuracy for identifying CI.^40^ These findings are consistent with our current study.

Because EC patients often presented symptoms in early stages of the disease, they tended to show low risk for lymph node metastasis. The clinical benefit of lymphadenectomy in the early stages is still controversial, and preoperative information about lymph node metastasis is essential for initial treatment planning. To minimize performing unnecessary lymphadenectomy in low‐risk group, Korean gynecologic oncology group proposed preoperative criteria to predict lymph node metastasis using preoperative CA‐125 level, presence of suspicious metastasis out of the uterine corpus and the depth of MI.^23^ Another scoring system, the LNM score, was proposed by Japanese group using tumor volume index, serum CA‐125 level, and tumor grade/histology.^22^ DWI could enhance the ability of MRI to detect metastatic lymph nodes by combining the size of node and relative ADC values.^41^ MRI showed a wide variation of sensitivity to detect lymph node metastases, ranging from 17% to 80%.^28^, ^42^, ^43^ Other imaging tools such as PET/CT and PET/MR did not significantly improve the detection of lymph node metastasis.^28^, ^43^ The NPVs in pelvic and/or para‐aortic nodal metastasis in this survey were 93.7% and 95.5%, respectively. There were 14 women of stage IIIC1 or IIIC2 with pelvic and/or para‐aortic lymph node underdiagnosed as stage I disease by preoperative MRI assessment. Eleven of them had less than three metastatic lymph nodes. And all of these 14 patients had only microscopic lymph node metastasis, which was a limitation of anatomic image study‐like MRI to detect the microscopic lymph nodal metastasis before surgery.

Adnexal and intra‐abdominal metastases are key components of EC staging. Information on the extent of the disease and intra‐abdominal metastasis, such as the presence of peritoneal or omental metastatic disease, are also important for the choice of surgical procedure. Intra‐abdominal metastasis including peritoneal and/or extrauterine metastases were a contraindication for laparoscopic surgery for EC patients. Some figures suggest ovarian metastasis, including bilateral ovarian involvement, morphological similarity between ovarian and uterine masses, and a larger uterine mass compared to the ovarian mass. A large unilateral ovarian mass or a low‐grade uterine mass without deep MI was seen to be a synchronous tumor.^44^ PET/CT has been demonstrated to be a useful tool in detecting metastatic deposits in the ovary, omentum, and distant spread.^31^, ^41^, ^45^ The sensitivity and specificity of MRI in detecting adnexal metastasis were 0% and 100% in previous reports.^41^, ^46^ The values of PPV and NPV for AM were 45.5% and 94.3% in this survey. Nine stage IIIA patients with adnexal metastasis were not detected by preoperative MRI assessment. Again, all of these nine patients were microscopic adnexal lesions with normal adnexal appearance. This suggests that adnexal metastasis could not be accurately detected by the MRI in this study. We noted that seven cases having tumor invasion to the uterine serosa. However, they were not easily detected by MRI. The findings emphasize the importance of careful inspection and palpation during surgery to detect the intra‐abdominal metastatic lesions.

The combination of T1‐weighted, T2‐weighted, and DWI MRI image could also detect the intra‐abdominal metastases. One prospective study reported that the sensitivity, specificity, PPV, and NPV were 64.6%, 98.6%, 86.1%, and 95.4% for detecting the distant metastatic disease of endometrial cancer by PET/CT, respectively.^45^ The sensitivity, specificity, PPV, and NPV of MRI for the detection of intra‐abdominal metastases in this survey were 91.7%, 99.0%, 68.8%, and 99.8%, respectively. And one of the five false‐positive intra‐abdominal metastatic patients was EC combined with retroperitoneal leiomyosarcoma.

The preoperative MRI showing highest accuracy rate was for the assessment of intra‐abdominal metastasis in stage IVB disease. The rates at which the preoperative MRI‐based clinical stage matched the postoperative surgical stage were 85.2% in stage IA, 51.9% in stage IB, 35.5% instage II, 5.3% in stage IIIA, 33.3% in stage IIIB, 28.6% in stage IIIC1, 64.3% in stage IIIC2, and 93.8% in stage IVB diseases. While, the accuracy rates of stage II, IIIA and IIIB, and IIIC1 were less than 50%. The small sample of these stages (stage II, IIIA and IIIB, and IIIC1) could contribute to the low decrease the accuracy rate of preoperative MRI. Furthermore, many of the stage II to stage IIIC1 diseases such as cervical stromal or vaginal invasion, and adnexal or pelvic lymph node metastasis were microscopic lesions which could be missed by MRI.

A strength of our study is that a large number of patients were recruited. All images were interpreted by qualified and well‐experienced radiologists for the routine MRI examination of endometrial cancer. We applied several statistical methods to calculate inter‐rater reliability which was not performed in one previous study. The retrospective design of our study represents the major limitation and may introduce selection bias. We only recruited patients who underwent preoperative MRI at our hospital. It could be a limitation related to bias toward greater consistency in radiology reports. Another limitation was that not all patients underwent pelvic and para‐aortic lymph node dissection, which decreased the statistical power.

In conclusion, our results indicated that preoperative MRI had good correlations with the pathologic parameters and stages of endometrial endometrioid carcinoma. Preoperative MRI had high NPVs for disease extent, particularly the extrauterine spread. Preoperative MRI could provide an excellent tool for routine preoperative assessment of EC patients and is helpful to optimize the surgical approach. Surgeons should be aware of the pitfalls in each parameter in preoperative MRI image to decrease the discrepancies in clinical and pathologic stages.

## ETHICS STATEMENT

This study was approved by the Institutional Research Ethics Committee at the National Taiwan University Hospital (approval no. 201905106RIND). All of the patients’ data were fully anonymized before we accessed them and the Research Ethics Committee waived the requirement for informed consent.

## CONFLICT OF INTEREST

No potential conflict of interest was disclosed.

## AUTHOR CONTRIBUTIONS

CY Wu and WF Cheng: Conception and design. CY Wu, IL Shih, and WF Cheng: Development of methodology; CY Wu, YC Chiang, YJ Tai, YL Chen, HC Hsu, CA Chen, and WF Cheng: Acquisition of data (provided animals acquired and managed patients, provided facilities, etc.); CY Wu and WF Cheng: Analysis and interpretation of data (e.g., statistical analysis, biostatistics, computational analysis); CY Wu and WF Cheng: Writing the manuscript. CY Wu, YC Chiang, IL Shih, YJ Tai, YL Chen, HC Hsu, CA Chen, and WF Cheng: Review and revision of the manuscript.

## Supporting information

Figure S1

Table S1

Table S2

## Data Availability

The data that support the findings of this study are available upon request from the corresponding author. The data are not publicly available due to privacy or ethical restrictions.
