# Supplementary figures and images for: Preoperative magnetic resonance imaging predicts clinicopathological parameters and stages of endometrial carcinomas
Source: Cancer Med. 2021 Dec 30;11(4):993–1004. doi: 10.1002/cam4.4486 (PMC8855918; doi:10.1002/cam4.4486)

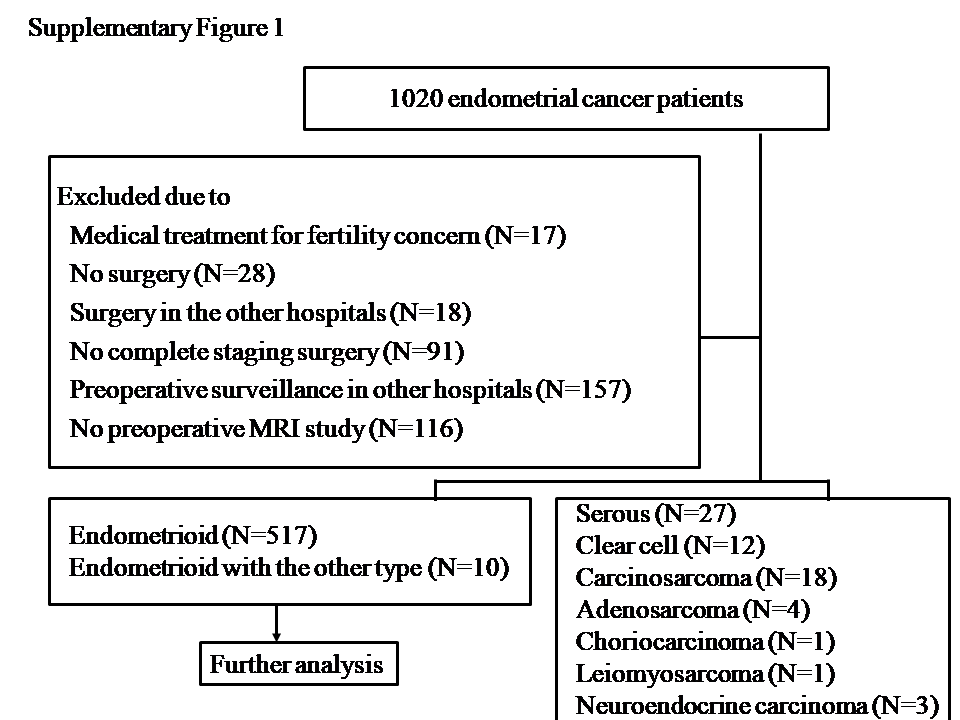

Supplement: Supplementary file 1 — Figure S1 [file CAM4-11-993-s001.tif]
