# Supplementary material for: Preoperative magnetic resonance imaging predicts clinicopathological parameters and stages of endometrial carcinomas
Source: Cancer Med. 2021 Dec 30;11(4):993–1004. doi: 10.1002/cam4.4486 (PMC8855918; doi:10.1002/cam4.4486)
Supplement: Supplementary file 2 — Table S1 [file CAM4-11-993-s002.docx]

**Table 1A.** The measures of the reliability of MRI in 294 EEC women by one radiologist

|  | Myometrial invasion >50% | Cervical stromal invasion | Adnexal involvement | Intra-abdominal metastasis | Pelvic nodal metastases | Para-aortic nodal metastases |
| --- | --- | --- | --- | --- | --- | --- |
| *Accuracy rate (%) | 85.4 | 91.8 | 93.9 | 99 | 88.5 | 92.5 |
| p | 0.0014 | 0.73 | 0.11 | 0.29 | 0.49 | 0.88 |
| Sensitivity (%) | 61.8 | 52.6 | 41.2 | 60 | 57.7 | 77.8 |
| Specificity (%) | 93.6 | 97.7 | 97.1 | 99.7 | 91.6 | 94.8 |
| Overestimation (%) | 4.8 | 2 | 2.7 | 0.3 | 7.6 | 4.5 |
| Underestimation (%) | 9.9 | 6.1 | 3.4 | 0.6 | 3.8 | 3 |

EEC: endometrial endometrioid carcinoma, N/A: not available, *: by Z-test

**Table 1B.** The measures of the reliability of MRI in 233 EEC women by the other 10 radiologists

|  | Myometrial invasion >50% | Cervical stromal invasion | Adnexal involvement | Intra-abdominal metastasis | Pelvic nodal metastases | Para-aortic nodal metastases |
| --- | --- | --- | --- | --- | --- | --- |
| *Accuracy rate (%) | 74.3 | 91 | 90.1 | 97.9 | 86.5 | 91.8 |
| Sensitivity (%) | 59.7 | 54.2 | 13.6 | 58.3 | 46.4 | 57.1 |
| Specificity (%) | 81.4 | 95.2 | 98.1 | 100 | 92.1 | 96.3 |
| Overestimation (%) | 12.5 | 4.3 | 1.7 | 0 | 7 | 3.3 |
| Underestimation (%) | 13.3 | 4.7 | 8.2 | 2.2 | 6.5 | 4.9 |

EEC: endometrial endometrioid carcinoma
