# Supplementary material for: Preoperative magnetic resonance imaging predicts clinicopathological parameters and stages of endometrial carcinomas
Source: Cancer Med. 2021 Dec 30;11(4):993–1004. doi: 10.1002/cam4.4486 (PMC8855918; doi:10.1002/cam4.4486)
Supplement: Supplementary file 3 — Table S2 [file CAM4-11-993-s003.docx]

**Supplementary Table 2A.** The measures of the reliability of MRI at 2013 in 80 EEC women

|  | Myometrial invasion >50% | Cervical stromal invasion | Adnexal involvement | Intra-abdominal metastasis | Pelvic nodal metastases* | Para-aortic nodal metastases* |
| --- | --- | --- | --- | --- | --- | --- |
| *Accuracy rate (%) | 86.3 | 95 | 93.8 | 98.8 | 82.3 | 92.9 |
| p | 0.11 | 0.37 | 0.43 | 0.24 | 0.074 | 0.65 |
| Sensitivity (%) | 64.3 | 50 | 40 | N/A | 60 | 50 |
| Specificity (%) | 90.9 | 98.7 | 97.3 | 98.8 | 83.8 | 100 |
| Overestimation (%) | 7.5 | 1.3 | 2.5 | 1.3 | 15.2 | 0 |
| Underestimation (%) | 6.3 | 3.6 | 3.8 | 0 | 2.5 | 7.1 |

EEC: endometrial endometrioid carcinoma, N/A: not available, *: by Z-test

**Supplementary Table 2B.** The measures of the reliability of MRI at 2018 in 95 EEC women

|  | Myometrial invasion >50% | Cervical stromal invasion | Adnexal involvement | Intra-abdominal metastasis | Pelvic nodal metastases* | Para-aortic nodal metastases* |
| --- | --- | --- | --- | --- | --- | --- |
| *Accuracy rate (%) | 76.8 | 91.6 | 90.5 | 95.8 | 91.4 | 96.2 |
| Sensitivity (%) | 53.6 | 54.6 | 11.1 | 50 | 63.6 | 75 |
| Specificity (%) | 86.6 | 96.4 | 98.8 | 100 | 95.1 | 100 |
| Overestimation (%) | 9.5 | 3.2 | 1.1 | 0 | 4.3 | 0.3 |
| Underestimation (%) | 13.7 | 5.3 | 8.4 | 4.2 | 4.3 | 3.9 |

EEC: endometrial endometrioid carcinoma
